# Two Single Nucleotide Polymorphisms in the Purinergic Receptor *P2X7* Gene Are Associated with Disease Severity in Multiple Sclerosis

**DOI:** 10.3390/ijms232315381

**Published:** 2022-12-06

**Authors:** Franca Rosa Guerini, Cristina Agliardi, Elisabetta Bolognesi, Milena Zanzottera, Domenico Caputo, Maria Barbara Pasanisi, Marco Rovaris, Mario Clerici

**Affiliations:** 1 IRCCS Fondazione Don Carlo Gnocchi, ONLUS, 20148 Milan, Italy; 2 Pathophysiology and Transplantation Department, University of Milan, 20122 Milan, Italy

**Keywords:** multiple sclerosis, multiple sclerosis severity score, purinergic receptor, P2X7 receptor polymorphisms

## Abstract

Multiple sclerosis (MS) is a chronic autoimmune inflammatory disease of the central nervous system (CNS) that leads to progressive physical disability. Recent evidence has suggested that P2X7 receptor (P2X7R)-mediated purinergic signalling pathways play a role in MS-associated neuroinflammation, possibly contributing to disease pathogenesis. To evaluate possible associations between *P2X7R* polymorphisms and MS disease severity, we performed an association study of five non-synonymous SNPs coding variants of the *P2X7R* gene: rs1718119 Ala348Thr, rs2230911 Thr357Ser, rs2230912 Gln460Arg, rs3751143 Glu496Ala, and rs28360457 Arg307Gln, modulating P2X7R expression in 128 MS patients (relapsing remitting MS, RRMS: *n* = 94; secondary progressive, SPMS: *n* = 34). All patients were genotyped, and multiple sclerosis severity score (MSSS) was evaluated in every case; 189 healthy subjects were enrolled as well as controls. Results showed that *P2X7R* rs1718119(A) 348Thr and rs22390912(G) 464Arg, two SNPs of minor allele frequency (MAF) known to confer gain of function to the P2X7R protein, were associated with significantly higher MSSS in RRMS patients alone (SMRR (*p* < 0.001, *p* = 0.01, respectively)). Interestingly, two whole haplotypes resulted in having significant association with MSSS in these same patients. Thus: (1) the *P2X7R-4* “ACGAG” haplotype, characterized by the co-presence of the rs1718119-rs2230912 AG MAF alleles, was associated with higher MSSS (Beta: 1.11 *p* = 0.04), and (2) the *P2X7R-1* “GCAAG” complementary haplotype, which contains the rs1718119 and rs2230912 GA wild-type alleles, was more frequently carried by patients with lower MSSS and less severe disease (Beta: −1.54 *p* < 0.001). Although being preliminary and needing confirmation in an ampler cohort, these results suggest that 348Thr and 464Arg variants have a role as modulators of disease severity in RRMS patients.

## 1. Introduction

Multiple sclerosis (MS) is a chronic autoimmune inflammatory disease of the central nervous system (CNS) that mainly affects young adults and leads to progressive physical disability [1]. The aetiology of such a condition is still unknown, although genetic, hormonal, latitude, and viral infections have been identified as risk factors [2] of MS onset. The disease is characterized by glial cell activation and infiltration of peripheral immune cells, resulting in the development of focal demyelinated lesions in the white and the grey matter of the cortex, the basal ganglia, the brain stem, and the spinal cord, with subsequent diffuse neurodegeneration [3]. From a clinical standpoint, different MS phenotypes can be recognized. Thus, clinically isolated syndrome (CIS) refers to the first clinical episode suggestive of demyelination of the CNS, while radiologically isolated syndrome (RIS) characterizes individuals who present with incidental brain MRI findings similar to those observed in patients with MS but who clinically have no signs of MS. Patients with CIS or RIS are at high risk of developing either relapsing–remitting MS (RRMS), the most common form of the disease, or primary progressive MS (PPMS). Notably, over time RRMS often evolves into secondary progressive MS (SPMS), a progressive disability with or without superimposed relapses [4,5].

Activation of the purinergic receptor P2X7 (P2X7R) is believed to be deleterious in autoimmune diseases and was recently hypothesized to play a role in the pathogenesis of MS [6,7]. Thus, P2X7R is a major driver of inflammation [8,9], playing an important role in the activation of T lymphocytes, mast cells, macrophages, dendritic cells, and polymorphonuclear neutrophilic granulocytes [10,11,12]. In the CNS, in particular, P2X7R is expressed by microglia, and its activation contributes to the release of interleukin 1 β (IL1β), a potent pro-inflammatory cytokine. P2X7R is an ATP-gated non-selective cationic channel that allows the passage of K^+^, NA^+^, and Ca^+^ through the cell membrane, and its activation can be driven by high concentrations of ATP and leads to the generation of large, cytolytic conductance pores [13,14]. Notably, besides its pore-forming effect, P2X7R activation can also result in apoptosis as a consequence of the activation of the caspase cascade via P2X7R-dependent stimulation of the NLRP3 inflammasome [15,16].

A number of non-synonymous single nucleotide polymorphisms (SNPs) have been identified in the gene encoding the human P2X7R on chromosome 12q24.3. In particular, four SNPs (rs1718119(G>A) Ala348Thr, rs2230911(C>G) Thr357Ser, rs2230912(A>G) Gln460Arg, and rs3751143(A>C) Glu496Ala), which form a haplotype block spanning exons 11 to 13 of the *P2X7R* gene, were suggested to modulate P2X7R expression and the subsequent secretion of IL1β [17]. Interestingly, the minor allele variant rs1718119(G>A) Ala348thr, together with rs2230912(A>G) Gln464Arg, was reported to result in a gain of receptor function, thus influencing a proinflammatory condition associated with inflammatory disorders such as rheumatoid arthritis, Sjogren’s syndrome, and systemic juvenile idiopathic arthritis [18]. Conversely, the rs2230911(C>G) Thr357Ser and the rs3751143(A>C) Glu496Ala variants were suggested to confer a reduced receptor function [19,20].

Recent results showed that glial cells expressing purinergic P2X7R are involved in the initial pathological mechanisms of disease, playing a role in experimental autoimmune encephalomyelitis (EAE) as well [21]. Thus, evidence from experimental studies using the EAE model of MS showed that microglia cells induce neuroinflammation via a P2X7R-dependent mechanism. Additional support to the possible role played by P2X7R in MS stems from the observation that rs28360457 (G>A) SNP coding for the Arg307Gln variant in the haplotype blocks *P2X7R* gene [22,23], resulting in reduced receptor function, which has been shown to associate with protection against MS risk [22].

In the attempt to shed further light on the role played by P2X7R in MS pathogenesis, we evaluated the possible correlations between *P2X7R* SNPs and haplotypes and clinical parameters of MS severity.

## 2. Results

### 2.1. Clinical Assessment

Demographic and clinical data of the 128 MS patients (34 SPMS and 94 RRMS) and the 189 healthy controls (HCs) enrolled in the study are summarized in Table 1. HCs were older, as per selection criteria, (54.3 ± 13.2) than both SPMS (32.3 ± 10.8) and RRMS (30.4 ± 10.1) (*p* < 0.001) patients to reduce enrolling false negative individuals as HCs.

Not surprisingly, multiple sclerosis severity score (MSSS) was significantly higher in SPMS (7.0 ± 2.2) compared to RRMS (3.6 ± 2.7) patients (*p* < 0.001); expanded disability status scale (EDSS), as well as disease duration, was higher in SPMS than in RRMS (*p* < 0.01). HLA-DRB1*15 was significantly more expressed in overall MS patients (<0.001) compared to HCs, confirming the association between the disease and this allele.

### 2.2. P2X7R Polymorphisms

All the individuals enrolled in the study were genotyped for the following *P2X7R* SNPs: rs1718119(G>A) Ala348Thr, rs2230912(A>G) Gln464Arg, rs2230911(C>G) Thr357Ser, rs3751143(A>C) Glu496Ala, and rs28360457 (G>A) Arg307Gln. Genotype distribution showed that all the variants were in Hardy–Weinberg equilibrium in each group, and no statistical difference in distribution between groups was evidenced (Supplementary Table S1).

Possible correlations between *P2X7R* SNPs genotypes, gender, age at disease onset, disease duration, and MSSS were evaluated next in all MS patients. A significant correlation between rs1718119 (G>A) Ala348Thr (*p* = 0.02) and higher MSSS values was evidenced in the overall group of MS patients. To summarize: (1) higher MSSS values were observed in patients carrying the minor allele frequency (MAF) rs1718119 (A):(AA+AG) compared to those carrying the major allele homozygous genotype (GG) (*p* = 0.005); and (2) higher MSSS scores were seen in patients carrying the MAF rs2230912(G):(GG+AG) genotype compared to those carrying the major allele homozygous genotype (AA) (*p* = 0.03) (Table 2).

Correlations between MSSS scores and *P2X7R* genotypes were then analysed upon dividing MS patients into those with a diagnosis of either SPMS or RRMS. Results indicated the presence of a statistical association between rs1718119(A) and rs2230912(G) MAF and higher MSSS scores in RRMS (*p* < 0.001 and *p* = 0.01, respectively) but not in SPMS patients.

In particular, significantly higher MSSS scores were observed in RRMS patients carrying the MAF rs1718119(A) than in those carrying the homozygous (GG) genotype (*p* < 0.001); similarly, rs2230912 (G)-carrying RRMS patients were characterized by higher MSSS scores compared to those carrying the (AA) genotype (*p* = 0.01). No associations between *P2X7R* variants and MSSS were observed in patients with a diagnosis of SPMS (Table 2). Finally, no association of *P2X7R* variants with gender nor with age at onset and disease duration was evidenced in the overall group of MS patients. 

A forward stepwise binary logistic regression analysis was finally performed to evaluate the possible association of MAF rs1718119(A) and rs2230912(G) (imputed as dependent variables) and MSSS after correction for factors known to influence disease severity, including age at onset, disease duration, DRB1*15 positivity, and gender, which were inputted as covariates. Significant associations were confirmed for rs1718119 (A) (*p* ≤ 0.01; OR:1.2, 95%CI:1.0–1.4 and *p* < 0.001; OR:1.1, 95%CI:1.2–1.8, respectively) and rs2230912(G) (*p* = 0.04; OR:1.2, 95%CI: 1.0–1.3 and *p* = 0.01; OR: 1.3, 95%CI: 1.1–1.5) and MSSS scores in the overall group of MS patients and in those with a diagnosis of RRMS. Again, no association was observed in SPMS patients.

### 2.3. Haplotype Analyses

**Figure 1:**
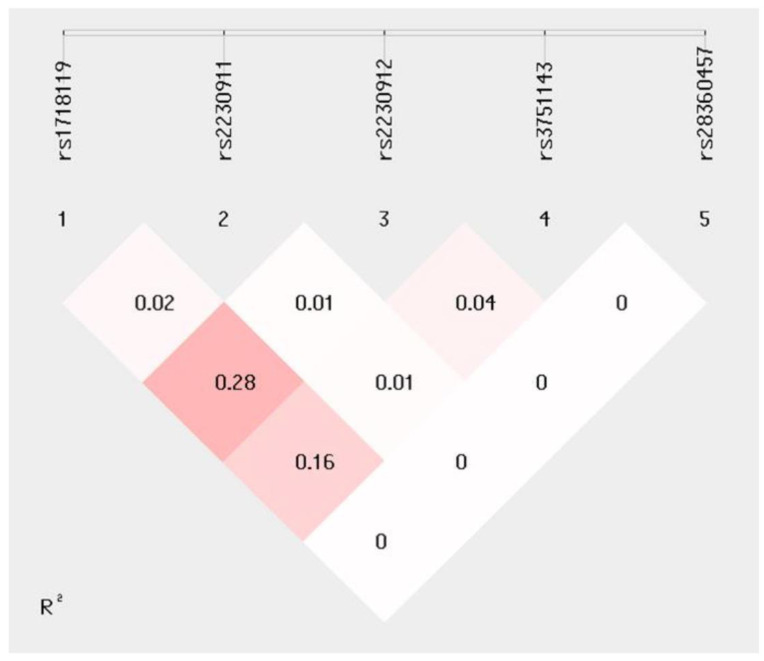
Linkage disequilibrium analysis (R^2^) for five *P2X7R* SNPs: rs1718119, rs2230911, rs2230912, rs3751143, and rs28360457.

Haplotype analyses were performed next to evaluate the possible presence of a linkage disequilibrium in SNPs between MS patients and HCs. A moderate R^2^ = 0.28 linkage disequilibrium was observed between rs1718119 (G>A) and rs2230912(A>G) (Figure 1). Haplotype distribution in MS and HCs and the association between MS haplotypes with MSSS values are summarized in Table 3. Haplotype frequency of distribution did not reveal any skewing between MS patients and HCs (Table 3).

Analysis of haplotype correlation with MSSS scores showed that the rs1718119(G>A), rs2230911(C>G), rs2230912(A>G), rs3751143(A>C), and rs28360457 (G>A) “GCAAG” called *P2X7R*-1 haplotype has a possible protective effect against MS severity. Thus, this haplotype was significantly associated with lower MSSS values both in the overall group of patients (Beta = −0.94 *p* = 0.01) and in RRMS patients (Beta: −1.54 *p* < 0.001). Notably, the *P2X7R*-4 “ACGAG” haplotype was instead weakly associated with higher MSSS in RRMS patients alone (Beta: 1.11 *p* = 0.04).

Once again, no significant correlation emerged between any haplotype and MSSS values observed in SPMS patients.

## 3. Discussion

Herein, we performed an association study of five non-synonymous SNPs coding functional variants of the *P2X7R* gene in relationship with MS development and disease severity. MS severity was assessed by MSSS, a disease-stratification method that combines disability scores (EDSS) and disease duration, and scores are calculated based on the distribution of EDSS within groups of MS patients of similar disease duration. Results showed that RRMS patients carrying the MAF of rs1718119(A) coding for a threonine in position 348 and the MAF of rs22390912 (G) coding for an arginine at position 464, two SNPs suggested to confer a gain of function to the P2X7R protein, had higher MSSS than those carrying the major alleles (coding for alanine and glycine, respectively). Because patients with low MSSS tend to progress at slower rates than those with higher MSSS, these data indicate that increased P2X7R activity is associated to more severe disease in RRMS. Notably, the association of MAF of rs1718119(A) and rs22390912(G) with higher MSSS was confirmed by regression analysis that included age of disease onset, gender, and the major genetic risk factor for MS onset, HLA-DRB1*15, as covariates. No correlations were evidenced between MSSS and the other three *P2X7R* SNPs (rs2230911 Thr357Ser, rs2836045 Arg307Gln, and rs28360457 Arg307Gln) we examined; these SNPs were suggested to confer loss of receptor function. Finally, our results did not confirm the possible protective role of rs28360457 Arg307Gln against MS development [22], probably due to the very low percentage of subjects carrying this variant.

In contrast with the results obtained in RRMS, no stratification of MSSS values in relationship with *P2X7R* polymorphisms was evidenced in patients with a diagnosis of SPMS. This discrepancy is likely the consequence of the fact that in this group of patients the disease severity, as evaluated by MSSS, was very high in all cases or, alternatively, could be due to the small number of SPMS patients analysed. It is nevertheless important to underline that, in comparison to RRMS, the extent of inflammation is lower in patients with SPMS, in whom degeneration prevails over inflammation [24]. RRMS is characterized by alterations of the blood–brain barrier (BBB) damage and by extensive infiltration of peripheral immune cells into the cerebrospinal fluid (CSF): autoreactive T and B lymphocytes, as well as monocytes, dendritic cells, and natural killer T cells, are seen in the CSF and concur in generating the neuroinflammatory milieu that plays a pivotal role in the pathogenesis of MS. As the disease progresses, the infiltration of peripheral immune cells through the BBB declines, and the inflammatory reaction is maintained only by microglia and astrocytes. P2X7R is the major actor in triggering and maintaining inflammation and in activating immune cells, leading to the release of IL1β, a potent proinflammatory cytokine. The biological role of this receptor being geared toward inflammation thus could explain the observation that *P2X7R* SNPs, resulting in protein gain of function, are associated with higher MSSS in RRMS alone, a stage of the disease that is primarily driven by inflammation. Notably, an increased expression of P2X7R was observed in demyelinating plaques adjacent to blood vessels within activated microglial cells/macrophages of post mortem collected tissues from SPMS patients [25] and in circulating monocytes of RRMS patients during disease relapse.

Since the *P2X7R* SNPs we analysed were suggested to be in linkage disequilibrium [17], haplotype segregations and their association with MSSS were evaluated too. Results confirmed the presence of moderate linkage disequilibrium between rs1718119 and rs22390912 SNPs, but no difference in haplotype distribution, was observed between patients and controls. Two whole haplotypes resulted nevertheless to be significantly associated with disease severity. Thus: (1) the *P2X7R*-4 “ACGAG” haplotype, which sees the co-presence of rs1718119-rs2230912 AG MAF alleles, was associated with a higher MSSS, and (2) the *P2X7R*-1 “GCAAG” complementary haplotype, which contains the rs1718119 and rs2230912 GA wild-type alleles, was more frequently carried by patients with lower MSSS and a less severe course of the disease. Analyses of whole haplotypes are important because of the co-presence of polymorphisms responsible for either gain (rs1718119 Ala348Thr and rs2230912 Gln464Arg) or loss of function (rs2230911 Thr357Ser, rs3751143 Glu496Ala, and rs28360457 Arg307Gln), which may counterbalance the effect of each other. It is noteworthy to underline that the *P2X7R*-4 “ACGAG” haplotype we observed to be associated with higher MSSS and more severe MS disease includes the haplotype previously shown to result in higher IL-1β production by monocytes, which could be completely suppressed by selective P2X7R antagonists [17].

P2X7R activation is suggested to be present in MS, and P2X7R-mediated purinergic signalling pathways are suspected to play a role in MS-associated neuroinflammation, possibly contributing to the pathogenesis of MS. This possibility was recently reinforced by results indicating that pharmacological blockade of P2X7R by brilliant blue G (BBG), its selective antagonist, delays the onset of the disease and alleviates clinical symptoms in EAE [26]. These results led to the suggestion that P2X7R should be considered as a target for a personalised therapeutic intervention in MS [21], with the potential of slowing down disease progression at least in the first phase of the disease.

A limitation of this study is represented by the limited number of SPMS patients and by the lack of patients with a diagnosis of primary progressive disease (PPMS). To this end, a replication study focusing on a larger cohort of MS patients with different disease phenotypes is planned. Analyses of possible correlations between *P2X7R* genotype and immune parameters in relationship with disease severity would be useful extensions of the current study. Finally, because RRMS can but does not always evolve into SPMS over time, it will be interesting to verify in longitudinal studies whether the presence of the gain-of-function *P2X7R* polymorphisms Arg464 and Thr348 SNPs will be able to predict which RRMS patients will develop SPMS.

## 4. Materials and Methods

### 4.1. Patients and Controls

A total of 128 patients with a diagnosis of multiple sclerosis according to McDonald’s revised diagnostic criteria [27] were enrolled (mean age at onset ± standard deviation (SD): 30.9 ± 10.3) at the Multiple Sclerosis Unit of IRCCS Fondazione Don Gnocchi ONLUS in Milano, Italy. Thirty-four of these patients had a diagnosis of SPMS, whereas 94 were diagnosed as being affected by RRMS disease. MSSS was calculated for both RRMS and SPMS patients. MSSS is a valid parameter of disability rate, taking into consideration both EDSS and disease duration [28]. One hundred and eighty-nine additional subjects were enrolled as HCs among the hospital staff. Exclusion criteria for HCs were the presence of neurological and/or autoimmune disorders. Moreover, since the average age of MS diagnosis globally is 32 years old [1], only HCs older than 34 years (54.3 ± 13.2) were enrolled to reduce the risk of analysing false healthy controls. Demographic data of patients and controls are reported in Table 1. All the subjects were characterized for HLA-DRB1*15 positivity, at present the strongest genetic risk factor for MS [29].

Informed consent was obtained from all the individuals before inclusion in the study. The study was conducted according to the guidelines of the Declaration of Helsinki and was approved by the institutional review board of the Don Carlo Gnocchi ONLUS Foundation, Milan (protocol number: #11_27/06/2019).

#### 4.1.1. Samples Collection and DNA Extraction

Whole blood was collected in 3 mL EDTA-containing vacutainer tubes (Becton Dickinson Co., Rutherford, NY, USA); genomic DNA was extracted from blood using standard phenol/chloroform procedures. Phenol chloroform extraction was performed by inducing cell lysis and DNA release with sodium dodecylsulfate (SDS) and proteinase K. Next, a phenol/chloroform/isoamyl alcohol mixture was added to the cell lysate to separate the proteins from the DNA [30]. The amount of DNA present in each sample was determined by measuring the optical density at 260 nm wavelengths using a Nanovue spectrophotometer (GE Healthcare, Chicago, IL, USA). DNA samples were stored at −20 °C until use.

#### 4.1.2. P2X7R SNPs Genotyping

*P2X7R* SNPs were evaluated by allelic discrimination real-time PCR using pre-designed TaqMan™ probes (Thermo Fisher Scientific, Waltham, MA, USA), in particular C_11704039_10 for rs1718119, C_15853705_20 for rs2230911, C_15853715_20 for rs2230912, C_27495274_10 for rs3751143, and C__59964938_10 for rs28360457. PCR consisted of a hot start at 95 °C for 10 min followed by 40 cycles of 94 °C for 15 s and 60 °C for 1 min. Fluorescence detection took place at 60 °C. Assays were performed in 10 μL reactions using 1 μL of DNA at 50 ng/μL, using TaqMan Genotyping Master Mix on 96-well plates using a CFX96 instrument (BioRad, Hercules, CA, USA). Control samples representing all possible genotypes and negative control were included in each reaction.

#### 4.1.3. HLA-DRB1*15 Characterization

The presence of the HLA-DRB1*15 allele, either in heterozygous or homozygous form, was inferred by the genotyping of the tag SNP rs3135388 [31] by allelic discrimination real-time PCR with the TaqMan™ C__27464665_30 probe and following the procedure described above.

#### 4.1.4. Statistical Analysis

Chi-square analysis was applied to verify that populations were in Hardy–Weinberg equilibrium. Genotype and MAF comparisons between groups were performed by chi-square evaluation in 3X2 and 2X2 tables of contingency. Two-sided *p* values after Bonferroni’s correction (pc) for the degree of freedom (df) were calculated, and the significance threshold was set at *p* < 0.05. ANOVA comparisons were performed to correlate *P2X7R* SNP genotypes and MAF association with MSSS. Binary logistic forward stepwise regression analysis was performed to evaluate the association of MAF rs1718119(A):(AA+AG) and rs2230912(G):(GG+AG) (imputed as dependent variables) and MSSS values after correction for factors that may influence MS severity such as age at onset, disease duration, DRB1*15 positivity, and gender, which were calculated as covariates. The odds ratio (OR) and its 95% confidence interval (CI) were used to measure the MAF association with the clinical scores. Statistical analyses were performed using SPSS software (v.28, IBM, in Armork, NY, USA).

Haplotype analysis of distribution and MSSS association were performed by Shesis plus online software (http://analysis.bio-x.cn, (accessed on 1 October 2022)) [32,33] and plink (gPLINK released under GNU GPLv2, http://pngu.mgh.harvard.edu/~purcell/plink/ (accessed on 1 October 2022)) software vs 2.020 [34].

## 5. Conclusions

These results confirm that P2X7R plays a role in RRMS severity and suggest that P2X7R could be considered as a target to design personalised therapeutic and rehabilitative intervention protocols in MS. Specifically, evidence herein allows us to speculate that the *P2X7R* 348Thr and 464Arg variants have a role as modulators of disease severity in MS patients, which deserves to be further investigated.

## Figures and Tables

**Table 1:** Demographic and clinical data of MS patients with secondary chronic progressive disease course (SPMS), patients with relapsing–remitting MS (RRMS), and healthy controls (HCs).

		Patients				
		SPMS *n* = 34	RRMS *n* = 94	Total MS *n* = 128	HCs *n* = 189	*p* Value
GENDER M/F: N		15/19	31/63	46/82	54/135	ns
AGE AT ONSET § years: mean ± SD		* 32.3 ±10.8	° 30.4 ±10.1	^ 30.9 ± 10.3	^§^ 54.3 ±13.2	^*° ^vs. §^ < 0.001
AGE AT MSSS years: mean ± SD		* 53.1 ±9.9	° 47.3 ±9.5	48.8 ± 9.9		* ^vs.^ ° < 0.01
Disease duration years: mean ± SD		* 22.7 ±7.7	° 17.9 ±9.5	19.2 ± 9.3		* ^vs.^ ° < 0.01
MSSS: mean ± SD		* 7.0 ±2.2	° 3.6 ±2.7	4.5 ± 2.9		* ^vs.^ ° < 0.001
DRB1*15 pos: N (%)		7 (20.6)	° 28 (29.8)	^ 35(27.3)	^§^ 24 (12.7)	^° ^vs. §^ < 0.001

§: for HCs age at enrolment; *: SPMS; °: RRMS; ^: total MS; ^§^: HCs; MSSS: multiple sclerosis severity score.

**Table 2:** *P2X7R* genotype distribution was reported in MS patients as N: absolute number and %: percentage, and multiple sclerosis severity score (MSSS) values were reported as mean and standard deviation (SD). ANOVA *p* value correlation analysis was reported between genotype, minor allele frequency (MAF) distribution, and MSSS values. Statistically significant associations are marked in bold.

			MSSS				MSSS				MSSS		
			SPMS (*n* = 34)				RRMS (*n* = 94)				Total (*n* = 128)		
P2x7R SNPs		N (%)	Mean	SD	*p* Value	N (%)	Mean	SD	*p* Value	N (%)	Mean	SD	*p* Value
rs1718119 Ala348Thr	GG	12 (35.3)	7.2	2.5		35 (37.2)	2.3	1.7		47 (36.7)	3.5	2.9	
	AG	16 (47.1)	6.7	2.1		44 (46.8)	4.5	2.8		60 (46.9)	5.1	2.8	
	AA	6 (17.6)	7.5	1.6	0.56	15 (16.0)	3.9	2.8	**<0.001**	21 (16.4)	5	3.0	**0.02**
MAF	**AA + AG**		6.8	1.9	0.59		4.4	2.8	**<0.001**		5.0	2.8	**0.005**
rs2230911 Thr357Ser	**CC**	32 (94.1)	7.1	2.1		84 (89.3)	3.7	2.7		116 (90.6)	4.6	2.9	
	CG	2(5.9)	4.9	2.5	0.16	10 (10.6)	3.0	2.2	0.47	12 (9.4)	3.3	2.3	0.15
rs2230912 Gln464Arg	AA	25 (73.5)	6.9	2.1		71 (74.7)	3.2	2.4		95 (74.2)	4.2	2.8	
	AG	9 (26.5)	6.9	2.5		20 (21.1)	5.1	3		30 (23.4)	5.7	2.9	
	GG	0			0.99	4 (4.2)	3.1	3.8	**0.01**	3 (2.4)	3.1	3.8	**0.03**
MAF	**GG + AG**		6.9	2.1	0.99		4.8	3.1	**0.01**		5.4	3.1	**0.03**
rs3751143 Glu496Ala	AA	24 (70.6)	7.0	2.3		55 (58.5)	3.2	2.5		79 (61.7)	4.3	3.0	
	AC	8 (23.5)	6.3	1.9		36 (38.3)	4.2	2.8		44 (34.4)	4.6	2.7	
	CC	2 (5.9)	8.8	0.3	0.33	3 (3.2)	3.6	2.2	0.16	5 (3.9)	5.7	3.3	0.59
MAF	**CC + AC**		6.8	2.0	0.74		4.2	2.7	0.06		4.7	2.8	0.48
rs2836045 Arg307Gln	GG	33(97.1)	6.9	2.2		92 (97.9)	3.6	2.7		125 (97.7)	4.4	2.9	
	AG	1 (2.9)	8.2	/	0.59	2 (2.1)	3.7	0.6	0.94	3 (2.3)	5.2	2.6	0.67

**Table 3:** *P2X7R* SNP frequencies (freq) of haplotype distribution in total MS patients, MS patients with secondary chronic progressive disease course (SPMS), MS patients with relapsing–remitting MS (RRMS), and healthy controls (HCs). Haplotype association with multiple sclerosis severity score (MSSS) values in total MS patients, RRMS, and SPMS subgroups was reported by Beta value. * *p* = 0.04; °° *p* < 0.001; § *p* = 0.01. Statistically significant associations are marked in bold.

HAPLOTYPE ASSOCIATION WITH MSSS												
SPMS *n* = 34	RRMS *n* = 94	Total MS *n* = 128	HCs *n* = 189	Haplotype	rs1718119	rs2230911	rs2230912	rs3751143	rs2836045	SPMS	RRMS	Total MS
freq	freq	freq	freq							Beta value	Beta value	Beta value
0.41	0.32	0.34	0.35	*P2X7R*-1	**G**	C	**A**	A	G	−0.05	**°°** **−1.54**	**§** **−0.94**
0.24	0.25	0.25	0.21	*P2X7R*-2	A	C	A	A	G	0.29	0.53	0.51
0.18	0.22	0.21	0.21	*P2X7R*-3	G	C	A	C	G	0.08	0.87	0.45
0.13	0.14	0.14	0.16	*P2X7R*-4	**A**	C	**G**	A	G	−0.07	* **1.11**	0.81
0	0.05	0.04	0.05	*P2X7R*-5	G	G	A	A	G	/	−0.68	−1.9
0	0.01	0.01	0.01	*P2X7R*-6	G	C	A	A	A	/	0.4	1.02
0.01	0	0.004	0.01	*P2X7R*-7	A	C	A	A	A	1.5	/	/
0.03	0	0.01	0.01	*P2X7R*-8	A	G	G	A	G	−2.12	/	/

## Data Availability

The data presented in this study are available upon request from the corresponding author.

## Supplementary Materials

The following supporting information can be downloaded at: https://www.mdpi.com/article/10.3390/ijms232315381/s1.

## References

[1] Walton C., King R., Rechtman L., Kaye W., Leray E., Marrie R.A., Robertson N., La Rocca N., Uitdehaag B., van der Mei I. (2020). Rising prevalence of multiple sclerosis worldwide: Insights from the Atlas of MS, third edition. Mult. Scler..

[2] Tarlinton R.E., Martynova E., Rizvanov A.A., Khaiboullina S., Verma S. (2020). Role of Viruses in the Pathogenesis of Multiple Sclerosis. Viruses.

[3] Lopes Pinheiro M.A., Kooij G., Mizee M.R., Kamermans A., Enzmann G., Lyck R., Schwaninger M., Engelhardt B., de Vries H.E. (2016). Immune cell trafficking across the barriers of the central nervous system in multiple sclerosis and stroke. Biochim. Biophys. Acta..

[4] Lassmann H. (2018). Multiple Sclerosis Pathology. Cold Spring Harb. Perspect. Med..

[5] Barizzone N., Leone M., Pizzino A., Kockum I., Martinelli-Boneschi F., D’Alfonso S., Multiple MS Consortium (2022). A Scoping Review on Body Fluid Biomarkers for Prognosis and Disease Activity in Patients with Multiple Sclerosis. J. Pers. Med..

[6] Burnstock G. (2017). Purinergic Signalling and Neurological Diseases: An Update. CNS Neurol. Disord. Drug Targets.

[7] Domercq M., Matute C. (2019). Targeting P2X4 and P2X7 receptors in multiple sclerosis. Curr. Opin. Pharmacol..

[8] Qian Y., Qian C., Xie K., Fan Q., Yan Y., Lu R., Wang L., Zhang M., Wang Q., Mou S. (2021). P2X7 receptor signaling promotes inflammation in renal parenchymal cells suffering from ischemia-reperfusion injury. Cell Death Dis..

[9] Rotondo J.C., Mazziotta C., Lanzillotti C., Stefani C., Badiale G., Campione G., Martini F., Tognon M. (2022). The Role of Purinergic P2X7 Receptor in Inflammation and Cancer: Novel Molecular Insights and Clinical Applications. Cancers.

[10] Di Virgilio F., Dal Ben D., Sarti A.C., Giuliani A.L., Falzoni S. (2017). The P2X7 Receptor in Infection and Inflammation. Immunity..

[11] Rivas-Yanez E., Barrera-Avalos C., Parra-Tello B., Briceno P., Rosemblatt M.V., Saavedra-Almarza J., Rosemblatt M., AcunaCastillo C., Bono M.R., Sauma D. (2020). P2X7 Receptor at the Crossroads of T Cell Fate. Int. J. Mol. Sci..

[12] Karmakar M., Katsnelson M.A., Dubyak G.R., Pearlman E. (2016). Neutrophil P2X7 receptors mediate NLRP3 inflammasome dependent IL-1beta secretion in response to ATP. Nat. Commun..

[13] Surprenant A., Rassendren F., Kawashima E., North R.A., Buell G., Muragaki Y., Mundlos S., Upton J., Olsen B.R. (1996). The Cytolytic P2Z Receptor for Extracellular ATP Identified as a P2X Receptor (P2X7). Science.

[14] Di Virgilio F., Chiozzi P., Falzoni S., Ferrari D., Sanz J.M., Venketaraman V., Baricordi O.R. (1998). Cytolytic P2X purinoceptors. Cell Death Differ..

[15] Savio L.E.B., de Andrade M.P., da Silva C.G., Coutinho-Silva R. (2018). The P2X7 Receptor in Inflammatory Diseases: Angel or Demon?. Front. Pharmacol..

[16] Kopp R., Krautloher A., Ramírez-Fernández A., Nicke A. (2019). P2X7 Interactions and Signaling—Making Head or Tail of It. Front. Mol. Neurosci..

[17] Stokes L., Fuller S.J., Sluyter R., Skarratt K.K., Gu B.J., Wiley J.S. (2010). Two haplotypes of the P2X(7) receptor containing the Ala-348 to Thr polymorphism exhibit a gain-of-function effect and enhanced interleukin-1beta secretion. FASEB J..

[18] Church L.D., Cook G.P., McDermott M.F. (2008). Primer: Inflammasomes and interleukin 1beta in inflammatory disorders. Nat. Clin. Pract. Rheumatol..

[19] Shemon A.N., Sluyter R., Fernando S.L., Clarke A.L., Dao-Ung L.P., Skarratt K.K., Saunders B.M., Tan K.S., Gu B.J., Fuller S.J. (2006). A Thr357 to Ser polymorphism in homozygous and compound heterozygous subjects causes absent or reduced P2X7 function and impairs ATP-induced mycobacterial killing by macrophages. J. Biol. Chem..

[20] Gu B.J., Zhang W., Worthington R.A., Sluyter R., Dao-Ung P., Petrou S., Barden J.A., Wiley J.S. (2001). A Glu-496 to Ala polymorphism leads to loss of function of the human P2X7 receptor. J. Biol. Chem..

[21] Sidoryk-Węgrzynowicz M., Strużyńska L. (2021). Astroglial and Microglial Purinergic P2X7 Receptor as a Major Contributor to Neuroinflammation during the Course of Multiple Sclerosis. Int. J. Mol. Sci..

[22] Gu B.J., Field J., Dutertre S., Ou A., Kilpatrick T.J., Lechner-Scott J., Scott R., Lea R.A., Taylor B.V., Stankovich J. (2015). A rare P2X7 variant Arg307Gln with absent pore formation function protects against neuroinflammation in multiple sclerosis. Hum. Mol. Genet..

[23] Miller C.M., Boulter N.R., Fuller S.J., Zakrzewski A.M., Lees M.P., Saunders B.M., Wiley J.S., Smith N.C. (2011). The Role of the P2X7 Receptor in Infectious Diseases. PLoS Pathog..

[24] Revesz T., Kidd D., Thompson A.J. (1994). A comparison of the pathology of primary and secondary progressive multiple sclerosis. Brain.

[25] Amadio S., Parisi C., Piras E., Fabbrizio P., Apolloni S., Montilli C., Luchetti S., Ruggieri S., Gasperini C., Laghi-Pasini F. (2017). Modulation of P2X7 Receptor during Inflammation in Multiple Sclerosis. Front. Immunol..

[26] Illes P. (2020). P2X7 Receptors Amplify CNS Damage in Neurodegenerative Diseases. Int. J. Mol. Sci..

[27] Thompson A.J., Banwell B.L., Barkhof F., Carroll W.M., Coetzee T., Comi G., Correale J., Fazekas F., Filippi M., Freedman M.S. (2018). Diagnosis of multiple sclerosis: 2017 revisions of the McDonald criteria. Lancet Neurol..

[28] Kalincik T., Kister I., Bacon T.E., Malpas C.B., Sharmin S., Horakova D., Kubala-Havrdova E., Patti F., Izquierdo G., Eichau S. (2022). Multiple Sclerosis Severity Score (MSSS) improves the accuracy of individualized prediction in MS. Mult. Scler..

[29] Jersild C., Fog T., Hansen G.S., Thomsen M., Svejgaard A., Dupont B. (1973). Histocompatibility determinants in multiple sclerosis, with special reference to clinical course. Lancet.

[30] Maniatis T., Fritsch E.F., Sambrook J. (1989). Molecular Cloning: A Laboratory Manual.

[31] de Bakker P.I., McVean G., Sabeti P.C., Miretti M.M., Green T., Marchini J., Ke X., Monsuur A.J., Whittaker P., Delgado M. (2006). A high-resolution HLA and SNP haplotype map for disease association studies in the extended human MHC. Nat. Genet..

[32] Shi Y.Y., He L. (2005). SHEsis, a powerful software platform for analyses of linkage disequilibrium, haplotype construction, and genetic association at polymorphism loci. Cell Res..

[33] Li Z., Zhang Z., He Z., Tang W., Li T., Zeng Z., He L., Shi Y. (2009). A partition-ligation-combination-subdivision EM algorithm for haplotype inference with multiallelic markers: Update of the SHEsis (http://analysis.bio-x.cn). Cell Res..

[34] Purcell S., Neale B., Todd-Brown K., Thomas L., Ferreira M.A., Bender D., Maller J., Sklar P., de Bakker P.I., Daly M.J. (2007). PLINK: A tool set for whole-genome association and population-based linkage analyses. Am. J. Hum. Genet..

