# Supplementary material for: Two Single Nucleotide Polymorphisms in the Purinergic Receptor P2X7 Gene Are Associated with Disease Severity in Multiple Sclerosis
Source: Int J Mol Sci. 2022 Dec 6;23(23):15381. doi: 10.3390/ijms232315381 (PMC9736555; doi:10.3390/ijms232315381)
Supplement: Supplementary file 1 [file ijms-23-15381-s001.zip › ijms-2057481-supplementary.pdf]

Table S1: Genotype distribution in MS and healthy controls.

		Patients		Healthy Controls	
		SPMS N=34	RRMS N=94	N=189	p value
		N (%)	N (%)	N (%)	
rs1718119 Ala348Thr:	GG	12 (35,3)	35 (37,2)	74 (39,2)	
	AG	16 (47,1)	44 (46,8)	90 (47,6)	
	AA	6 (17,6)	15 (16,0)	25 (13,2)	
					0,95
rs2230911 Thr357Ser:	CC	32 (94,1)	84 (89,3)	167 (88,4)	
	CG	2(5,9)	10 (10,6)	23 (11,6)	
					0,61
rs2230912 Gln464Arg:	AA	25 (73,5)	70(74,5)	132 (69,8)	
	AG	9 (26,5)	21 (22,3)	51 (27,0)	
	GG	0	3 (3,2)	6 (3,2)	
					0,88
rs3751143 Glu496Ala:	AA	24 (70,6)	55 (58,5)	114 (60,3)	
	AC	8 (23,5)	36 (38,3)	70 (37,0)	
	CC	2 (5,9)	3 (3,2)	5 (2,7)	
					0,52
rs2836045 Arg307Gln:	GG	33(97,1)	92 (97,9)	187 (98,9)	
	AG	1 (2,9)	2 (2,1)	2 (1,1)	
					0,63
